# Interfacial Fracture Energy Between New Translucent Zirconias and a Resin Cement

**DOI:** 10.3290/j.jad.b2916403

**Published:** 2022-04-13

**Authors:** Laura Patricia Ortiz Nadal, Nathália de Carvalho Ramos, João Paulo Mendes Tribst, Lilian Costa Anami, Renata Marques de Melo, Marco Antonio Bottino

**Affiliations:** a MSc Student, Department of Dental Materials and Prosthodontics, São Paulo State University (UNESP), Institute of Science and Technology, São José Dos Campos, SP, Brazil. Performed the experiments in partial fulfillment of requirements for a degree, wrote the manuscript.; b Postdoctoral Fellow, Department of Dental Materials and Prosthodontics, São Paulo State University (UNESP), Institute of Science and Technology, and also Professor at Department of Dentistry, University of Taubaté (UNITAU), Taubaté, SP, Brazil. Idea, hypothesis, experimental design, performed the interfacial fracture test, wrote the manuscript, proofread the manuscript.; c Assistant Professor, Department of Dental Materials, Academic Centre for Dentistry Amsterdam (ACTA), University of Amsterdam and Vrije Universiteit Amsterdam, Amsterdam, The Netherlands. Idea, hypothesis, experimental design, performed the finite element analysis, wrote the manuscript, proofread the manuscript.; d Dentist, Department of Dental Materials and Prosthodontics, São Paulo State University (UNESP), Institute of Science and Technology, São José Dos Campos, SP, Brazil. Experimental design, contributed substantially to discussion, proofread the manuscript.; e Researcher, Department of Dental Materials and Prosthodontics, São Paulo State University (UNESP), Institute of Science and Technology, São José Dos Campos, SP, Brazil. Idea, hypothesis, experimental design, contributed substantially to discussion, proofread the manuscript.; f Professor at Department of Dental Materials and Prosthodontics, São Paulo State University (UNESP), Institute of Science and Technology, São José Dos Campos, SP, Brazil. Idea, contributed substantially to discussion, proofread the manuscript.

**Keywords:** zirconia, finite element analysis, fracture, dental materials

## Abstract

**Purpose:**

To determine the interfacial fracture energy (IFE) and stress distribution of Brazil-nut–shaped specimens made of translucent zirconia and resin cement.

**Materials and Methods:**

Three types of translucent zirconia were used: 3Y-TZP (high, Vita YZ HT), 4Y-TZP (super, Vita YZ ST), and 5Y-TZP (extra, Vita YZ XT). The adhesive surfaces were air abraded and 10-MDP-based resin cement was used. The cemented Brazil-nut–shaped specimens, with an elliptical defect in the center (as in real Brazil nuts), were thermally aged (5°C–55°C; 40,000 cycles). The IFE test was conducted with a piston to apply compression on the specimen, while the adhesive interface was positioned at four different angles (0, 10, 20, and 30 degrees) to measure the IFE during tensile, shear, and mixed failure modes. All adhesive interfaces were observed to determine failure patterns. The finite element analysis (FEA) was used to calculate tensile and shear stress distributions according to inclinations. Statistical analysis was conducted using the Kruskal-Wallis and Dunn’s post-hoc tests (95%), as well as the Mann-Whitney test (95%) was applied to compare each group regarding the aging factor.

**Results:**

According to Kruskal-Wallis and Dunn’s post-hoc tests, there were no statistically significant differences between non-aged (p > 0.05) and aged materials (p > 0.05). However, there was a significant difference between aged and non-aged materials for all inclinations (p < 0.05) (Mann-Whitney test). According to the FEA, the compressive loading of Brazil-nut–shaped specimens at different angles showed a predominance of tensile stress at 0 degrees and shear stress at 30 degrees.

**Conclusion:**

The IFE under predominantly shear stresses is higher than when specimens are subjected only to tensile stresses, which allows the interpretation that failures in the oral environmental will probably occur preferentially under tensile stresses, because less energy is needed. All translucent zirconia bonded to resin cement has similar IFE, and thermal aging negatively affects these bonding interfaces.

Favorable characteristics, such as high biocompatibility, high hardness, chemical inertia, and improved esthetics provide good reasons for using zirconia-based ceramics as a replacement material for lost dental structures. However, adhesion to zirconia can be problematic, as shown by several reported surface treatments designed to improve zirconia bonding to resin cement, such as particle blasting and the use of 10-MDP-based primers, silanes, and/or resin cements.^7,10,17^ Therefore, evaluating the adhesive interface of zirconia restorations is necessary, considering that the adhesive strength of polycrystalline materials is not as predictable or high as that of vitreous materials.^15,16^

The mechanical behavior of zirconia can change as new generations develop. Modern translucent zirconia presents increased yttria content, a cubic phase, and varying-grain morphology.^20^ Thus, the ceramic structure affects the mechanics of bonding between restoration and the substrate.^8,12^ Moreover, more translucent zirconia has a lower grain size, flexural strength, and fracture toughness values than conventional zirconia and, because such a different microstructure critically influences adhesion, it requires further testing.

However, the information that frequently used adhesion tests can provide is limited.^13^ Susceptibility to interfacial fractures as a measure of bond quality is usually evaluated with (micro)tensile and (micro)shear tests. The results often depend on distributions of defects not characterized within the adhesive interfaces.^22^ Spurious stress distributions of the specimens during testing are also a disadvantage. Alternatively, interfacial fracture toughness is an inherent property that affects the resistance of an interface to crack propagation.^16^ Several methods using specimens of various shapes can determine interfacial fracture energy, such as double-cantilever beam (DCB) and notchless triangular prism (NTP) specimens, but only the “Brazil-nut” specimen geometry allows measuring interfacial fracture toughness in the full range of failure modes.^19,20^ Testing using Brazil-nut–shaped specimens measures interfacial fracture toughness on the complete scale of mixed forces, from pure mode I (pure tensile) to pure mode II (pure shear). The mixed mode associated with interfacial fracture is controllable by varying the phase angle. Hence, mixed-mode failures are also a better representation of what occurs during occlusal loading in the oral environment.^1,19,23^

This study aimed to determine interfacial fracture energy and susceptibility to interfacial aging of highly, super-, and extra-translucent zirconia bonded to resin cement, as well as to analyze the stress distribution in Brazil-nut–shaped specimens to understand the tensile, shear, and mixed failure modes. The null hypothesis of this study was that different types of zirconia would present similar interfacial fracture toughness, decreasing only after aging.

## Materials and Methods

### Specimen Preparation

**Fig 1:**
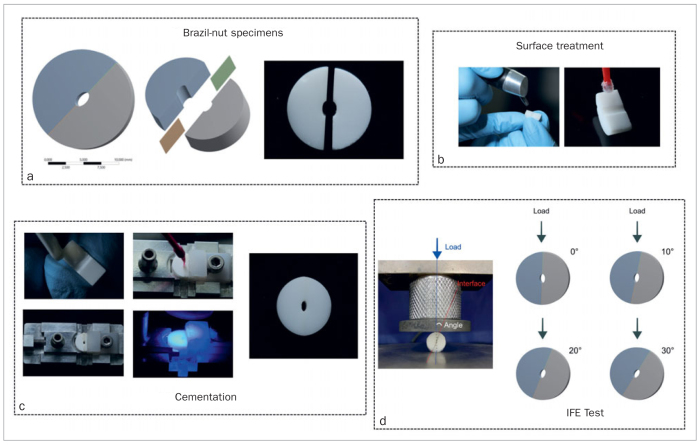
a. Brazil-nut-shaped specimens: computer-aided design of the specimens and the specimen milled in zirconia; b. surface treatments of the specimens: air-abrasion and silane application; c. cementation procedures: resin cement application, specimen positioned on cementation jig, excess removal, light curing, and the Brazil-nut specimen bonded; d. interfacial fracture test energy (IFE): compression test and schematic representation of load application and adhesive interface angle.

Blocks of highly translucent (HT) (3Y-TZP, Vita In-Ceram HT, Vita Zahnfabrik; Bad Säckingen, Germany), super-translucent (ST) (4Y-TZP, Vita In-Ceram ST, Vita Zahnfabrik), and extra-translucent (XT) (5Y-TZP, Vita In-Ceram XT, Vita Zahnfabrik) zirconia were machined in a pre-sintered form through lateral wear to a cylindrical shape with a diameter of 10 mm, and sectioned into 5-mm-thick disks in a precision cutter (IsoMet 1000 Precision Saw, Buehler; Lake Buff, IL, USA). The disks were cut in the middle also using the precision cutter to obtain equal halves. An internal elliptically shaped defect 2 mm in diameter (minor axis of the ellipse) was created in each hemi-disk using a fully automatic 5-axis milling machine to complete the Brazil-nut–shaped specimen (Fig 1a). The specimens were made with 20% larger pre-sintering dimensions to compensate for sintering shrinkage. After sintering, the diameter (minor axis) shrank to 1.5 mm.

The specimens were sintered in a Sirona inFire HTC speed oven (Dentsply Sirona; Charlotte, NC, USA) according to the manufacturer’s recommendations. The heating ratio and maintenance temperature used were 17°C/min and 1450°C for HT, 8°C/min and 1530°C for ST, and 4°C/min and 1450°C for XT. The maintenance time was 120 min for all groups.

**Fig 2:**
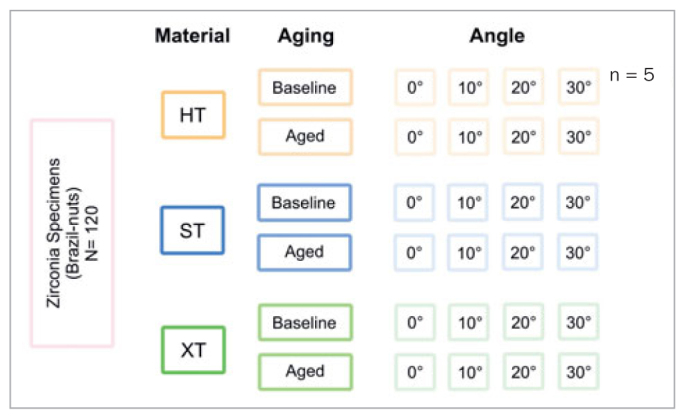
Design of the study showing the groups of materials, aging, and angles used during the interfacial fracture energy test. HT: highly translucent zirconia, 3Y-TZP; ST: super-translucent zirconia (4Y-PSZ); XT: extra-translucent zirconia (5Y-PSZ).

The specimens were divided into three groups according to material: HT, ST, and XT zirconia. Each group was subdivided into baseline (n = 20) and aged specimens (n = 20) (Fig 2).

### Cementation of Specimens

After sintering, the specimens were cleaned in an ultrasonic bath with distilled water. The specimens were then air abraded with 50-μm aluminum oxide particles at a distance of 1 cm and 2 bar pressure for 2 s/cm^2^. All samples were cleaned after sandblasting, the Clearfil Ceramic Primer (Kuraray Noritake; Tokyo, Japan) was actively applied (Fig 1b), and the hemidisks were cemented with 10-MDP-based resin cement (Panavia F 2.0, Kuraray Noritake) using a specific cementation device. Light curing was performed for 40 s using a light-curing unit with 1200 mW/cm^2^ and wavelengths of 385 to 515 nm (Bluephase N, Ivoclar Vivadent; Schaan, Liechtenstein) (Fig 1c).

The specimens were stored for 60 days in an incubator at 37°C and immersed in distilled water after cementation. Half of the specimens were aged thermally for 40,000 cycles (5°C–55°C), after which all were tested.^19^ The statistical power was calculated using the OpenEpi (Open Source Epidemiologic Statistics for Public Health, www.openepi.com) at a 95% confidence interval. A power of 89.9% was found necessary to compare the baseline values, while 91.0% was necessary to compare the aged groups.

In accordance with Ramos et al,^19^ the interfacial fracture test was conducted in a universal test machine (Emic DL-1000, Emic; São José dos Pinhais, PR, Brazil) with a load cell of 1000 kgf for the compression test (Fig 1d). The test was performed with a displacement speed of 0.001 mm/s, using angles (θ, crack slope towards the load application) of 0, 10, 20, and 30 degrees (Fig 1d). Five specimens of each ceramic type were tested at each angle. The critical load for interface fracture was used to calculate the energy release rate, G (interfacial fracture energy).

The stress intensity factors corresponding to mode I (KI) and mode II (KII) were derived by adjusting polynomial forms using the pre-crack angle (θ), critical load (P) for interfacial detachment, and relative length of the pre-crack (a/R), in which 2a is the length of the pre-crack, and R is the Brazil-nut–shaped specimen’s radius.

The combination of modes I and II is characterized by the load phase Ư, which is controlled by the angle of the pre-crack, θ. The sample is under pure traction at 0 degrees, predominantly shear at 30 degrees, and in mixed modes at angles between these two values. The loading phase is calculated by the equation below:


$$\Psi =\tan^{-1}\left(\frac{K_{II}}{K_{I}}\right)$$

The critical condition for crack growth is given by the release rate (N/m), which is in critical energy when the interface debonds, given by the equation below:


$$Gc=G_{1}+G_{2}=\frac{1}{E_{1}}(K_{I}^{2}+K_{II}^{2})$$

where G1 and G2 are the energy release rates of modes I and II, respectively, and E1 is Young’s modulus in the plane strain state.

### Failure Analysis

After the interfacial fracture tests, all specimens were observed under a stereomicroscope (Discovery V20, Zeiss; Jena, Germany); representative specimens were observed using SEM (Inspect S50, FEI; Brno, Czech Republic) to characterize how the fracture occurred inside the interface between the zirconia and the resin cement. Only the failure data that occurred at the interface were statistically evaluated. Additional specimens were also observed using SEM to characterize the microstructure and surface grains.

The failures were classified as in Ramos et al,^19^ who described four types: type I: cement is found on both sides of the specimens; type II: one side of the interface was covered with cement and the other side had no remnants on zirconia, the crack started on one side of the interface and ran on the same side; type III: traces of cement on both sides, and the crack starts on one interface and kinks to the other one; type IV (kinking); there are cement clusters over the entire adhesive surface area.

### Stress Distribution (FEA)

To calculate stress distribution according to the adhesive area angle during the compression test, a three-dimensional (3D) finite element analysis (FEA) was applied. Therefore, a 3D model was obtained using the BioCAD protocol from a scanned image of the specimens, representing the Brazil-nut–shaped specimens. Thus, the model picture from a representative sample was captured in a stereomicroscope (Discovery V20, Zeiss) in lateral view and exported in BMP (Bitmap) to be used as the background in modeling software (Rhinoceros 6.0SR8, McNell North America; Seattle, WA, USA). The structures considered for modeling were zirconia and resin cement (80 µm) at the interface.

After modeling refinement, computer-aided engineering software was used to simulate the compressive load as in the in vitro test. Therefore, the STP (Standard for the Exchange of Product Data) was exported to the software (ANSYS 19R1, Ansys; Houston, TX, USA). Next, a three-dimensional analysis was selected to create coordinates. A geometric model was generated and the element meshes were created based on a convergence test (10%) using a tetrahedral element with hard behavior and soft transition.

The elastic moduli and Poisson’s ratio of each material were determined according to the previous literature,^9^ mainly to perform the static structural analysis. Each material was considered elastic, homogeneous, and isotropic. Thus, meshes controlled by quadratic tetrahedral elements were used.

For processing, the Brazil-nut–shaped specimen was considered fixed in all directions of the interface and a force of 500 N was applied to a contact area of 1 mm^2^ measured from the in vitro test. The relationship between surfaces determines the transmission of existing stresses from one element to another in the interface region. Thus, bonded contacts were considered for all relationships.

The adhesive interface was positioned at four different angles relative to the long axis of load application (0, 10, 20, and 30 degrees) according to the in vitro steps. Tensile and shear stresses were selected as criteria, and the results were plotted as colorimetric stress maps aided by a color scale, in which each shade represents a range of stresses generated in the structures evaluated. The stress peak of both analysis criteria at each load angle was plotted for quantitative comparison.

## Results

### Interfacial Fracture Energy

**Fig 3:**
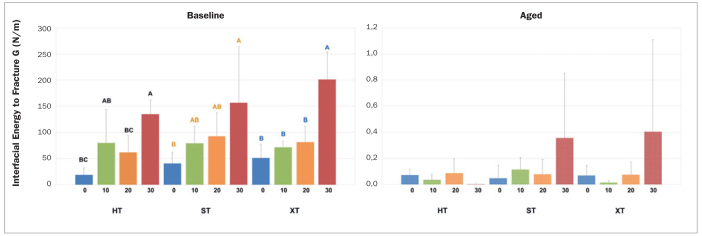
Interfacial fracture energy graph (N/m). Different letters indicate statistically significant differences among the angles for each ceramic (HT, ST, and XT). After aging, there were no statistically significant differences among the groups.

The Kolmogorov-Smirnov, Shapiro-Wilk, and Levene (95%) normality tests were performed, showing that the data did not present normal behavior and were not homoscedastic (p = 0.000). Thus, the Kruskal-Wallis and Dunn tests were performed, both at a 5% significance level. However, there were no statistically significant differences between the materials for non-aged (p_0_ = 0.485, p_10_ = 0.8580, p_20_ = 0.4027, and p_30_ = 0.650) and aged (p_0_ = 0.6976, p_10_ = 0.3505, p_20_ = 0.7565, and p_30_ = 0.1469) groups (Fig 3).

Additionally, the Mann-Whitney test was applied, also with a significance level of 5%, to compare each of the groups regarding the aging factor and the angles for each ceramic. A statistically significant difference between aged and non-aged materials at each angle was found (p > 0.05). Considering baseline groups, the loading phase (angles) was different for HT (p = 0.001), ST (p = 0.047), and XT (p = 0.000) zirconia, while this was not significant for aged groups (p > 0.05) (Fig 3).

Figure 3 shows the behavior of the groups at angles of 0 degrees (pure tensile) and 30 degrees (predominantly shear). Although the behavior tendency is the same, the values of interfacial fracture energy under shear stress are higher than when the samples are submitted only to tensile stress.

### Finite Element Analysis (FEA)

**Fig 4:**
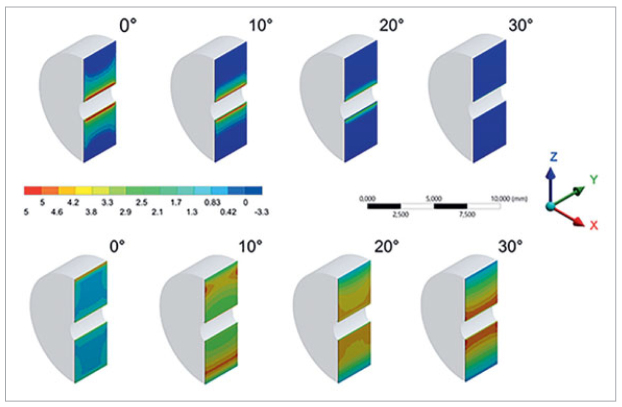
Stress generated on the adhesive interface according to the FEA. The upper Brazil-nut–shaped specimens indicate the tensile stress generated and the bottom row indicates shear stress at different angles.

Different response patterns were observed, in which the tensile stress in the cement (5.34, 4.87, 3.57, and 1.07 MPa) was inversely proportional to the angle of the load applied (0, 10, 20, and 30 degrees, respectively). The highest stress found was shear in most of the cement when the interface was positioned at 30 degrees (Fig 4).

Thus, the compressive loading of Brazil-nut–shaped specimens at different inclinations fosters the predominance of tensile or shear stresses, depending on the inclination angle (Fig 4).

### Fractographic Analysis

**Fig 5:**
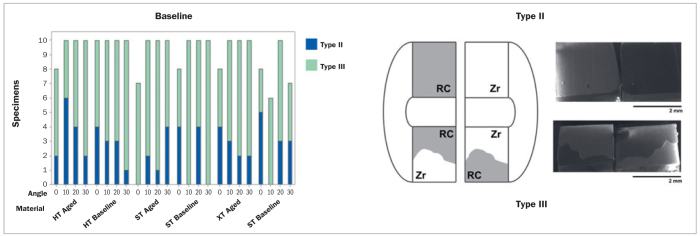
Failure type graph (left), schematic representation (center), and a representative micrograph of failure types (right), showing type II (all resin cement on one side) and type III (resin cement found on both sides) failures. RC indicates the area covered by resin cement and Zr indicates the area covered by zirconia.

The failures were classified as described elsewhere.^19^ In the present study, type II failures occurred, in which one side of the interface was covered with cement and the other side had no remnants on zirconia. The crack started on one side of the interface and ran on the same side. In type III failures, which showed traces of cement on both sides, the crack started on one interface and kinked to the other (Fig 5). Failure types I and IV were not observed in the present specimens.

**Fig 6:**
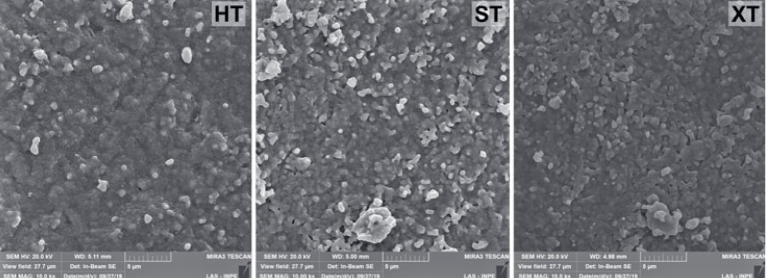
SEM images of translucent zirconia. Highly (HT), super- (ST), and extra- (XT) translucent.

Figure 6 shows the SEM images of the zirconia surface, which indicate a similar microstructure among them.

## Discussion

In the present study, the interfacial fracture energy that bonded 10-MDP-based resin cement to zirconia was evaluated using mixed-mode conditions with Brazil-nut–shaped specimens. The different levels of translucent zirconia (HT, ST, and XT) behaved similarly regarding fracture energy and failure modes, although there was a higher IFE at an angle of 30 degrees, at which shear stresses prevail. The null hypothesis was accepted, considering that the three tested translucent zirconias tested showed similar behavior regarding the immediate interfacial fracture energy, but the values decreased after thermal aging.

The Brazil-nut–shaped specimens have a standard elliptical defect in the center of the disk, which ensures crack initiation at the interface until complete fracture.^19,23^ According to the literature, this standard notch and an interface at several inclinations during the test allows measuring the IFE of the full range of failures between pure mode I (tensile) and mode II (shear).^19,23,24^ The present study corroborated that the defect allowed crack initiation/propagation throughout the adhesive interface, with higher values at higher angles.

The microstructure of more translucent zirconias (HT, ST, and XT) is similar (Fig 6), agreeing with Sen and Isler,^21^ but mechanical properties such as Vickers hardness, fracture toughness, biaxial flexural strength, and fatigue behavior are different.^2^ However, many differences between these ceramics were observed regarding interfacial fracture toughness. Therefore, the relationship between the microstructure of the material and crack propagation still needs clarification.

According to our results, thermal aging decreased interfacial fracture energy, even diminishing the differences between the different inclinations. This implies that regardless of stress type, the interface will break easily after hydrothermal degradation. Long-term water storage and thermocycling are the most commonly used methods to test the adhesive durability of resins and resin cements. Both tests are considered clinically relevant aging parameters and can modify the surface roughness and phase transformation of zirconia.^4^ The study by Guilardi et al^6^ showed that the fracture load of aged zirconia groups significantly decreased after fatigue, compared to non-aged groups, corroborating the results of this study. The authors suggest two explanations for this load decrease: first, adhesion becomes very weak after aging and therefore reduces the fatigue load of the assembly; secondly, the reduction might have been caused by the higher viscosity of this cement, which leads to a lower capacity to fill the defects created by the air-abrasion system. Both processes probably facilitated hydrolysis from the aging process (thermocycling + storage) to more quickly degrade the interface, breaking the adhesive chemical bonds, and resulting in hydrolytic cement degradation.

A study by Lüthy et al^14^ corroborated the fact that thermocycling affects the adhesive interface between resin cement and stabilized tetragonal zirconia. However, consensus is lacking in the literature on the number of cycles needed for degradation. In the present study, the samples were subjected to 40,000 cycles in baths of alternating temperatures of 5–55°C for 30 s each, which was an aggressive aging protocol compared to other studies, such as Guilardi et al,^6^ who performed 12,000 thermal cycles and already found a decrease in specimen strength. The protocol of this study was based on Ramos et al,^19^ who also performed 40,000 thermal cycles and used similar specimen geometries with a similar adhesive area.

**Table 1:** Materials used in this study, their manufacturers and composition

Material	Manufacturer	Composition
Zirconia 3Y-TZP – Vita YZ HT white	Vita Zahnfabrik; Bad Säckingen, Germany.	ZrO_2_: 90.4–94.5%; Y_2_O_3_: 4–6%; HfO_2_: 1.5–2.5%; Al_2_O_3_:0–0.3%; Er_2_O_3_: 0–0.5%; Fe_2_O_3_: 0–0.3%
Zirconia 4Y-PSZ – Vita YZ ST white	Vita Zahnfabrik	ZrO_2_: 88–93%; Y_2_O_3_: 6–8%; HfO_2_: 1–3%; Al_2_O_3_: 0–1%; pigments: 0–1%
Zirconia 5Y-PSZ – Vita YZ XT white	Vita Zahnfabrik	ZrO_2_: 86–91%; Y_2_O_3_: 8–10%; HfO_2_: 1–3%; Al_2_O_3_: 0–1%; pigments: 0–1%
Aluminum oxide 50 µm – 220 MESH	Bio-Art Equipamentos Odontológicos; São Paulo, Brazil	Al_2_O_3_: 95.50%; Fe_2_O_3_: 2.70%; CaO: 0.19%; K_2_O: 0.09%; MgO: 0.25%; TiO_2_: 2.70%; Na_2_O: 0.02%; SiO_2_: 0.90%
Clearfil Ceramic Primer	Kuraray Noritake; Tokyo, Japan	10-(phosphonooxy)decyl methacrylate (10-MDP), silane primer, γ-MPS, ethanol
Panavia F 2.0 Resin Cement	Kuraray Noritake	10-MDP, hydrophobic aromatic and aliphatic photoinitiator, dibenzoyl peroxide dimethacrylate, hydrophilic dimethacrylate, silanized silica

Regarding the surface treatments, air abrasion is the most indicated mechanical treatment for improving bond strength to the zirconia surface and increasing longevity, and it can be performed with different protocols, varying the type and size of particles, pressure, distance, and time of application. A surface treatment that induces minimum or no surface damage is preferable for the long-term success of bonded ceramic restorations.^6^ Although it depends on the type of zirconia used, the surface conditioning method for 5Y-PSZ differs from 3Y-TZP, because it cannot alter its properties in a controlled way in response to mechanical stresses. The crystallographic and morphological configuration of 5Y-PSZ prevents the toughening mechanism by phase transformation typical of 3Y-TZP, and is essential to limit the surface damage inevitably caused by airborne-particle abrasion.^5^

The resin cement used was 10-MDP-based, which improves zirconia bonding and is the best combination associated with sandblasting for cementation. It provides a chemical interaction between the hydroxyl groups of the zirconium oxide layer and the phosphate ester monomers of the 10-MDP in the resin cement at the interfacial level, meaning that each phosphate group (tridentate bridging mode: three zirconium atoms) binds to one, creating zirconium phosphate – a thermally and hydrolytically stable zirconium salt.^5^

Finite element analysis (FEA) mapped the specimens and interfaces according to stress types. Using a two-dimensional FEA, Chai et al^3^ determined the interfacial energy associated with delamination growth throughout the veneer/core interface, considering the presence of channel cracks.^3^ The present study showed that tensile stress in the cement layer was inversely proportional to the angle of the load applied (0, 10, 20, and 30 degrees). It was also determined that shear was the highest stress type found in most of the cement when the interface was positioned at 30 degrees. The same was observed in the fracture toughness test, and the results were possibly due to the difficulties in crack propagation, which increases crack growth resistance.^11^

As opposed to other studies using Brazil-nut–shaped specimens,^19,23,24^ this study showed that even at angles of 30 degrees, the interfaces are not subjected to pure shear. As the angle increases, shear stresses increase, but the tensile stress is not eliminated, causing the interface to be subjected to predominantly shear stresses. Therefore, further fracture-mechanics studies must investigate whether adhesive interfaces are subjected to pure shear stresses at some phase angle. Clinically, it is necessary to understand the behavior of the adhesive interface in other circumstances, such as in varying pH, and how to minimize the occurrence of interfacial failures according to the preparation and design of the restoration.

All failure types were adhesive (types II and III), contrary to the study by Ramos et al,^19^ which found cohesive failures in resin cement. However, these failures only occurred in glass-infiltrated zirconia groups, which reflects a much higher bond strength than conventional surface treatments of zirconia (air-borne particle abrasion). In the present study, zirconia was only abraded with aluminum oxide but the predominance of the type II failure mode shows that under mixed-mode conditions, the crack deflected towards the side of the bonded surface under tensile stress.^17^

According to previous reports, the tensile and shear stress criteria must be evaluated and can both be responsible for an increase in the adhesive failure risk when calculating higher magnitudes.^22^ Therefore, studies evaluating tensile and shear strengths are valid methods to measure bond strength in adhesively bonded zirconia restorations.^22^ However, the present study evaluated interfacial fracture toughness under pure tensile, mixed, and predominantly shear stress modes. This allows extrapolating the present results to a clinical scenario, meaning that the restorations in clinical practice would be more susceptible to failure under tensile stress, as the energy required for interface fracture is lower under tensile than shear stress. Clinically, tensile stress prevails in the cervical/proximal margins of the crowns.^18^

All in vitro studies have limitations, but this study was necessary to increase understanding of the fracture mechanics of the adhesive interface between resin cement and zirconia. Considering that interfacial fracture energy is an inherent property of such an interface, the authors believe a true bonding measure was obtained.

## Conclusion

Regardless of the type of translucent zirconia, interfacial fracture energy under predominantly tensile stress is lower than when specimens are subjected only to shear stress. Thermal aging negatively affected the adhesive interfaces, reducing interfacial fracture energy, thus increasing the risk of adhesive failure for adhesively luted translucent zirconia restorations, including tensile, mixed, or shear components.

## Clinical relevance:

Using interfacial fracture energy, this study showed that cement cohesive failures will probably occur mostly under tensile stress regions. Therefore, dentists should consider preparation designs and bonding procedures that could reduce the tensile stress areas when a zirconia-based ceramic has been elected as the restorative material in a harsh oral environment.
